# Impacts of the preceding cancer-specific health-related quality of life instruments on the responses to the subsequent EQ-5D-5L

**DOI:** 10.1186/s12955-022-02085-8

**Published:** 2023-01-17

**Authors:** Shoki Izumi, Yasuhiro Hagiwara, Yutaka Matsuyama, Takeru Shiroiwa, Naruto Taira, Takuya Kawahara, Keiko Konomura, Shinichi Noto, Takashi Fukuda, Kojiro Shimozuma

**Affiliations:** 1 Biostatistics and Bioinformatics Course, Graduate School of Interdisciplinary Information Studies, The University of Tokyo, Tokyo, Japan; 2 Department of Biostatistics, School of Public Health, The University of Tokyo, 7-3-1, Hongo, Bunkyo-ku, Tokyo, 113-0033 Japan; 3 Center for Outcomes Research and Economic Evaluation for Health, National Institute of Public Health, Wako, Japan; 4 Department of Breast and Endocrine Surgery, Okayama University Hospital, Okayama, Japan; 5 Clinical Research Promotion Center, The University of Tokyo Hospital, Tokyo, Japan; 6 Center for Health Economics and QOL Research, Niigata University of Health and Welfare, Niigata, Japan; 7 Department of Biomedical Sciences, College of Life Sciences, Ritsumeikan University, Kusatsu, Japan

**Keywords:** Cost-effectiveness analysis, EORTC QLQ-C30, EQ-5D-5L, FACT-G, Health-related quality of life, Order effect

## Abstract

**Background:**

In clinical studies, the EQ-5D-5L is often employed with disease-specific health-related quality of life instruments. The questions in the former are more general than the latter; however, it is known that responses to general questions can be influenced by preceding specific questions. Thus, the responses to the EQ-5D-5L have the possibility of being influenced by the preceding disease-specific health-related quality of life instruments. This may lead to bias in the cost-effectiveness analysis results. Therefore, this study aimed to evaluate the impact of the preceding cancer-specific health-related quality of life instruments on the EQ-5D-5L responses.

**Methods:**

We prepared questionnaire booklets containing the EQ-5D-5L, the European Organization for Research and Treatment of Cancer Quality of Life Questionnaire Core 30, and the Functional Assessment of Cancer Therapy General with different orders. Using a quasi-randomized design, they were distributed to the patients undergoing drug therapy for advanced cancer, who were classified into three groups: Groups 1, 2, and 3 (the EQ-5D-5L placed first, second, and last, respectively). We compared the EQ-5D-5L index and the missingness of EQ-5D-5L among the groups.

**Results:**

The mean EQ-5D-5L index was 0.796, 0.760, and 0.789 for groups 1 (n = 300), 2 (n = 306), and 3 (n = 331), respectively. The difference between Groups 2 and 1 was − 0.036 (95% CI − 0.065 to − 0.007; p = 0.015). The proportion of patients with an incomplete EQ-5D-5L was 0.11, 0.11, and 0.05 for Groups 1, 2, and 3, respectively. The difference of the proportions between group 3 and 1 and between 3 and 2 was − 0.06 (95% CI − 0.10 to − 0.02; p = 0.003) and − 0.06 (95% CI − 0.10 to − 0.02; p = 0.003), respectively.

**Conclusions:**

Although the EQ-5D-5L index differed according to the instrument orders, the difference size would not be considerably larger than the minimally important difference. The patients tended to complete the EQ-5D-5L when they were placed at the end of the questionnaire.

**Supplementary Information:**

The online version contains supplementary material available at 10.1186/s12955-022-02085-8.

## Introduction

Since the results of a cost-effectiveness analysis help to properly allocate limited medical resources, many countries utilize it for decision-making regarding healthcare systems [1, 2]. Thus, conducting it using reliable methods is important, for which several guidelines are available [3–9]. The quality-adjusted life year (QALY), weighting lifetime with health state utilities, is usually recommended as an outcome measure [3, 4, 8]. Furthermore, the EQ-5D index is often used as a health state utility [10]; it is a multi-attribute preference-based measure consisting of five dimensions [11]. Although previously only the three-level version of the EQ-5D was available, the one with five levels, the EQ-5D-5L, was developed for increasing sensitivity and decreasing the ceiling effect [12, 13].

The EQ-5D is frequently employed with disease-specific health-related quality of life (HRQOL) instruments in clinical studies, such as randomized controlled trials that often provide health utility information for health economic evaluation. The EQ-5D and the disease-specific HRQOL instruments are used simultaneously; this is because the former’s five items incompletely describe patients’ health status for clinical assessment due to the smallness of the number of items, while the latter can detect disease-specific symptoms and treatment impacts with high sensitivity. Additionally, the two are utilized concurrently in research developing mapping algorithms from the disease-specific HRQOL instruments onto the EQ-5D indexes [14].

However, theory of order effects and previous studies evaluating the order effects on the responses of the HRQOL instruments indicate the EQ-5D’s possible susceptibility to it. In this paper, order effects refer to the phenomenon that the different orders in which instruments or questions presented influence the responses to them. On the one hand, the order effects on HRQOL instruments consisting of many questions has been unobserved in three studies [15–18], and small differences in few subscales have been observed in another two [19, 20]. On the other hand, for those comprising one question, although a large sample size may be a cause, four studies have reported order effects [21–24]. Thus, the less questions the HRQOL instruments contain, the more susceptible their responses may be to the preceding instruments. This tendency is consistent with the theory of order effects; if the HRQOL instruments contain less questions the questions would be more general, and it is known that responses to general questions can be influenced by preceding specific questions [25]. Additionally, the responses to the EQ-5D correlate with the responses to the disease-specific HRQOL instruments [26–30]. Therefore, the EQ-5D, consisting of one item for each dimension, may be influenced by the HRQOL instrument orders, though direction of the effects was unpredictable. Although a study examining the order effects on the EQ-5D-3L index did not find a difference, this could be because it was continually preceded by the MOS 36-Item Short-Form Health Survey, which is an HRQOL measure consisting of 36 detailed questions [31]. Furthermore, thus far, no study has assessed the order effects on the EQ-5D-5L responses.

If instrument orders impact the EQ-5D-5L responses, the order effects could bias the cost-effectiveness analysis results. For example, when health state utilities are extracted from multiple studies in which different orders of the disease-specific HRQOL instruments and the EQ-5D-5L are adopted, the aforementioned analysis’ results may be influenced due to the order effects. In mapping research, the latter may or may not be preceded by disease-specific measures that may also bias mapping algorithms, resulting in biased results of the cost-effectiveness analysis.

This research investigated the impact of the preceding disease-specific HRQOL instruments on responses to the EQ-5D-5L in the field of oncology. Specifically, we focused on two common cancer-specific HRQOL instruments, namely, the European Organization for Research and Treatment of Cancer Quality of Life Questionnaire Core 30 (EORTC QLQ-C30) and the Functional Assessment of Cancer Therapy General (FACT-G) and evaluated the differences in the responses to the EQ-5D-5L’s five questions, its mean index, its correlations with the cancer-specific HRQOL instruments’ subscales, and the missingness of its index among the instrument orders.

## Methods

### Data collection

This research used data from Quality of Life Mapping Algorithm for Cancer (QOL-MAC) study’s data that mainly purported to develop mapping algorithms for the EORTC QLQ-C30 and the FACT-G on the EQ-5D-5L index. Its details have been published elsewhere [32]. The QOL-MAC study was conducted in accordance with the Declaration of Helsinki; the research protocol was approved by each participating hospital.

In the QOL-MAC research, patients with unresectable, locally advanced, recurrent, or metastatic cancers were recruited from 14 hospitals in Japan from November 2018 to March 2019. The eligible patients had lung, stomach, colorectal, breast cancer, or other solid tumors; they were aged 20 years or above and were undergoing drug therapy, with an Eastern Cooperative Oncology Group (ECOG) performance status of 0–3. Those who were receiving treatment for multiple primary tumors or were unable to respond to the questionnaires were excluded. All enrolled patients provided written informed consent before participating in the study.

We utilized those questionnaire booklets that contained the EQ-5D-5L, FACT-G, and EORTC QLQ-C30 in different orders; let us denote them as E, F, and Q, respectively. We created six types of questionnaire booklets, each of which contained the HRQOL instruments in any of the following orders: EFQ, EQF, FEQ, QEF, FQE, or QFE. For example, EFQ represents questionnaire booklets comprising the HRQOL instruments in the following order: EQ-5D-5L > FACT-G > EORTC QLQ-C30. We designed the booklets in such a manner that the medical staff could not identify these measures’ order by observing their covers.

We distributed the questionnaire booklets to the patients by a quasi-randomization design using the order in which patients enrolled; therefore, each patient was assigned to any of the six groups defined by its types. Firstly, we repeatedly lined the six questionnaire booklet types in a fixed order (specifically, EFQ > EQF > FQE > FEQ > QFE > QEF) and sent them to all hospitals. Subsequently, each hospital’s medical staff recruited the patients and distributed the questionnaire booklets in the aforementioned order. Finally, the patients answered the questionnaire booklets, which were principally collected in the hospital. When this was unfeasible, the patients returned them directly to the data center.

### The HRQOL instruments

The EQ-5D-5L is a multi-attribute preference-based measure comprising five questions: mobility, self-care, usual activities, pain/discomfort, and anxiety/depression [12]. Each item is rated on a five-point scale: no, slight, moderate, severe, and extreme problems (wording differs according to the items). We calculated the EQ-5D-5L index using the value sets for Japan, England, and the United States [33–35]; one and zero represented full health and death, respectively. The three value sets were used to serve to future studies using some of them.

The 30-item EORTC QLQ-C30 (version 3) is a cancer-specific HRQOL instrument [36]. It includes five functional subscales (physical, role, cognitive, emotional, and social functioning), a global health and quality-of-life subscale, three symptom subscales calculated using several questions (fatigue, nausea and vomiting, and pain), and six symptom items (dyspnea, insomnia, appetite loss, constipation, diarrhea, and financial difficulties). Each subscale score ranged from 0 and 100; it was calculated when half or more subscale questions were answered. High scores on the functional and global health and quality-of-life subscales denoted better health status, while those on the symptom subscales or items signified severe symptoms.

The FACT-G (version 4) is a cancer-specific HRQOL instrument containing 27 questions [37]. It comprises four subscales regarding well-being: physical, social/family, emotional, and functional. Each subscale’s score was computed when over half of its questions were answered. The FACT-G total score was calculated when more than 80% of all its questions were answered and all subscale scores were obtained. High subscale and FACT-G total scores represented better health status.

### Statistical analysis

The QOL-MAC study’s sample size was determined based on the main purpose (i.e., the mapping algorithms’ development) and feasibility. Hence, no sample size calculation for the order effects’ evaluation was conducted.

We employed two analysis populations: the eligible and the completed EQ-5D-5L. The former included all enrolled and suitable patients; it was used for the statistical analysis of the missing EQ-5D-5L indexes. The latter comprised all participating and eligible patients whose EQ-5D-5L indexes were calculated; it was utilized for the statistical analysis of the EQ-5D-5L items and index.

The patients were classified into three groups based on the EQ-5D-5L’s placement in the questionnaire booklets: Groups 1, 2, and 3 consisted of the two booklet types that had the EQ-5D-5L in the first, second, and last places, respectively. The differences in the responses of this scale’s each item between the groups were examined using the Wilcoxon rank sum test. Furthermore, the differences in the mean EQ-5D-5L indexes between the groups were estimated using analysis of variance with the EQ-5D-5L’s order as the only explanatory variable. Additionally, we conducted multivariable analysis and analysis with inverse probability weighting (IPW) (see Additional file 1 for details) [38]. Spearman rank correlation analysis between the EQ-5D-5L index and the subscales of EORTC QLQ-C30 and FACT-G were conducted (see Additional file 1).

Using a linear binomial regression (also called linear probability model) with the EQ-5D-5L’s order as the only explanatory variable, we compared the proportions of those patients whose EQ-5D-5L were incomplete due to some reason; who failed to return the questionnaire booklets to the data center; who submitted the questionnaire booklets unanswered; and who returned these booklets with some incomplete responses. Additional details regarding the statistical analysis including multivariable analysis and analysis of proportions of the FACT-G and the EORTC QLQ-C30 with missing subscales are provided in Additional file 1.

Each p-value was two-tailed, and p < 0.05 was considered to be nominally statistically significant. All statistical analyses were conducted using SAS software, version 9.4 (SAS Institute).

## Results

**Fig. 1:**
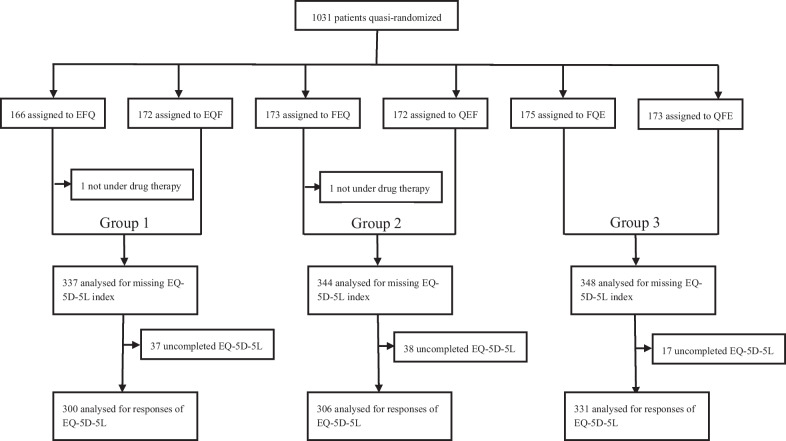
Patient flow chart. EFQ, EQF, FEQ, QEF, FQE and QFE respectively stand for the questionnaire type containing HRQOL instruments in the order of EQ-5D-5L > FACT-G > EORTC QLQ-C30, EQ-5D-5L > EORTC QLQ-C30 > FACT-G, FACT-G > EQ-5D-5L > EORTC QLQ-C30, EORTC QLQ-C30 > EQ-5D-5L > FACT-G, FACT-G > EORTC QLQ-C30 > EQ-5D-5L and EORTC QLQ-C30 > FACT-G > EQ-5D-5L. Groups 1, 2, and 3 consisted of the two questionnaire types that had EQ-5D-5L in the first, second, and last places, respectively. EORTC QLQ-C30, European Organization for Research and Treatment of Cancer Quality of Life Questionnaire Core 30; FACT-G, Functional Assessment of Cancer Therapy General

Overall, 1,031 patients were enrolled in the QOL-MAC study (Fig. 1), of which 2 were excluded due to eligibility criteria violations (not receiving drug therapy); thus, the eligible population comprised 1,029 patients. The completed EQ-5D-5L population included 937 patients, after excluding 92 patients. Their demographic and clinical characteristics were similar among the three groups (Table 1). The median ages of Groups 1, 2, and 3 were 68, 67, and 68, respectively; 54% were male. Furthermore, lung cancer was the most common (35%), followed by colorectal cancer (25%); 50% and 41% had an ECOG performance status of 0 and 1, respectively. The patient characteristics between those with complete and incomplete EQ-5D-5Ls have been shown in Additional file 1: Table S1.

**Table 1:** Demographic and clinical characteristics of the eligible patients with a completed EQ-5D-5L

Characteristic	Group 1 (N = 300)	Group 2 (N = 306)	Group 3 (N = 331)
Age (in years)	68 (58–75)	67 (58–73)	68 (59–74)
Sex			
Male	157 (52%)	163 (53%)	189 (57%)
Female	143 (48%)	143 (47%)	142 (43%)
Tumor type			
Lung cancer	108 (36%)	108 (35%)	113 (34%)
Stomach cancer	21 (7%)	22 (7%)	23 (7%)
Colorectal cancer	79 (26%)	73 (24%)	80 (24%)
Breast cancer	37 (12%)	35 (11%)	44 (13%)
Other solid tumors	55 (18%)	68 (22%)	71 (21%)
Stage at diagnosis			
I	12 (4%)	19 (6%)	18 (5%)
II	23 (8%)	17 (6%)	34 (10%)
III	63 (21%)	64 (21%)	65 (20%)
IV	200 (67%)	204 (67%)	206 (62%)
Unknown	2 (1%)	2 (1%)	8 (2%)
Site of metastasis or recurrence^a^			
None	26 (9%)	22 (7%)	35 (11%)
Liver	60 (20%)	72 (24%)	79 (24%)
Lung	110 (37%)	95 (31%)	91 (27%)
Bone	48 (16%)	54 (18%)	67 (20%)
Brain	34 (11%)	35 (11%)	31 (9%)
Lymph nodes	124 (41%)	129 (42%)	120 (36%)
Others	69 (23%)	73 (24%)	90 (27%)
History of surgery			
Yes	159 (53%)	152 (50%)	186 (56%)
No	141 (47%)	154 (50%)	144 (44%)
Unknown	0 (0%)	0 (0%)	1 (0%)
Hospitalization			
Yes	60 (20%)	71 (23%)	64 (19%)
No	240 (80%)	235 (77%)	267 (81%)
ECOG performance status			
0	156 (52%)	148 (48%)	160 (48%)
1	115 (38%)	127 (42%)	143 (43%)
2	21 (7%)	21 (7%)	25 (8%)
3	7 (2%)	10 (3%)	3 (1%)
Unknown (0, 1, 2, or 3)	1 (0%)	0 (0%)	0 (0%)
Type of treatment^a^			
Chemotherapy	186 (62%)	195 (64%)	225 (68%)
Endocrine therapy	20 (7%)	22 (7%)	25 (8%)
Molecular targeted therapy	56 (19%)	49 (16%)	58 (18%)
Immunotherapy	38 (13%)	43 (14%)	31 (9%)
Palliative therapy	17 (6%)	10 (3%)	10 (3%)
Others	3 (1%)	2 (1%)	3 (1%)
Adverse event at enrollment			
Yes	186 (62%)	200 (65%)	220 (66%)
No	113 (38%)	106 (35%)	110 (33%)
Unknown	1 (0%)	0 (0%)	1 (0%)

The median (IQR) and the number (%) were reported for age and other characteristics, respectively. Groups 1, 2, and 3 consisted of the two questionnaire types that had the EQ-5D-5L in the first, second, and last places, respectively

ECOG, Eastern Cooperative Oncology Group; IQR, interquartile range

^a^Multiple choices were allowed

Regarding mobility, the responses between Groups 1 and 2 and between 1 and 3 were significantly different (Table 2). Specifically, the proportion of the patients who reported “no problems” with mobility was smaller in Groups 2 (48%) and 3 (50%) than in Group 1 (65%), whereas the proportion of those with “slight problems” was larger in Groups 2 (29%) and 3 (30%) as compared to Group 1 (17%). Moreover, the responses to usual activities between Groups 1 and 2 and between 2 and 3 varied significantly. Specifically, the proportion of the patients who did not indicate problems with usual activities was smaller in Group 2 (40%) than in Groups 1 (50%) and 3 (50%), while that of those who reported slight difficulties was larger in Group 2 (40%), as compared to Groups 1 (31%) and 3 (29%). The results distinguishing FEQ and QEF in the questionnaire booklets that had the EQ-5D-5L in the second place are displayed in Additional file 1: Table S2.

**Table 2:** Responses to the EQ-5D-5L’s five items in the three groups

	Response level					P-value	
	1	2	3	4	5	vs Group 1	vs Group 2
Mobility							
Group 1	194 (65%)	51 (17%)	26 (9%)	23 (8%)	6 (2%)	–	–
Group 2	146 (48%)	88 (29%)	41 (13%)	21 (7%)	10 (3%)	< 0.001	–
Group 3	165 (50%)	100 (30%)	47 (14%)	17 (5%)	2 (1%)	0.004	0.310
Self-care							
Group 1	243 (81%)	28 (9%)	16 (5%)	8 (3%)	5 (2%)	–	–
Group 2	242 (79%)	40 (13%)	12 (4%)	5 (2%)	7 (2%)	0.657	–
Group 3	271 (82%)	42 (13%)	10 (3%)	6 (2%)	2 (1%)	0.606	0.322
Usual activities							
Group 1	150 (50%)	92 (31%)	31 (10%)	22 (7%)	5 (2%)	–	–
Group 2	122 (40%)	123 (40%)	32 (10%)	16 (5%)	13 (4%)	0.045	–
Group 3	167 (50%)	95 (29%)	43 (13%)	22 (7%)	4 (1%)	0.998	0.045
Pain/discomfort							
Group 1	122 (41%)	126 (42%)	39 (13%)	10 (3%)	3 (1%)	–	–
Group 2	118 (39%)	140 (46%)	31 (10%)	12 (4%)	5 (2%)	0.807	–
Group 3	136 (41%)	137 (41%)	40 (12%)	15 (5%)	3 (1%)	0.988	0.784
Anxiety/depression							
Group 1	188 (63%)	85 (28%)	16 (5%)	7 (2%)	4 (1%)	–	–
Group 2	169 (55%)	100 (33%)	24 (8%)	10 (3%)	3 (1%)	0.057	–
Group 3	206 (62%)	93 (28%)	22 (7%)	10 (3%)	0 (0%)	0.896	0.068

The number (%) was reported for each response level. Responses 1 to 5 correspond to no problems, slight problems, moderate problems, severe problems, and extreme problems, respectively (wording differs according to the items). Groups 1, 2, and 3 consisted of the two questionnaire types that had the EQ-5D-5L in the first, second, and last places, respectively

The mean EQ-5D-5L indexes based on the value set for Japan in Groups 1, 2, and 3 were 0.796 (95% CI 0.776–0.817), 0.760 (0.740–0.781), and 0.789 (0.769–0.808), respectively (Table 3); it was the lowest in Group 2. The difference between Groups 2 and 1 was − 0.036 (95% CI − 0.065, − 0.007; p = 0.015), and that between Groups 3 and 2 was 0.029 (0.000, 0.057; 0.049). The discrepancies based on the three value sets in the EQ-5D-5L index’s mean differences between the groups were less than 0.01 for all pairwise comparisons. The analysis adjusted for covariates and the analysis adjusted for the missing EQ-5D-5L indexes through the IPW showed similar results (Additional file 1: Tables S3 and S4). A forest plot for the subgroup analysis is shown in Additional file 1: Fig. S1. Additional file 1: Table S5 contains the results discriminating between FEQ and QEF.

**Table 3:** Mean EQ-5D-5L indexes in the three groups

	Mean (95% CI)	Difference (95% CI; P-value)	
		vs Group 1	vs Group 2
Japanese value set			
Group 1	0.796 (0.776, 0.817)	–	–
Group 2	0.760 (0.740, 0.781)	− 0.036 (− 0.065, − 0.007; 0.015)	–
Group 3	0.789 (0.769, 0.808)	− 0.008 (− 0.036, 0.021; 0.597)	0.029 (0.000, 0.057; 0.049)
England value set			
Group 1	0.821 (0.797, 0.844)	–	–
Group 2	0.791 (0.768, 0.814)	− 0.030 (− 0.063, 0.003; 0.071)	–
Group 3	0.822 (0.800, 0.844)	0.001 (− 0.031, 0.033; 0.948)	0.031 (− 0.001, 0.063; 0.056)
US value set			
Group 1	0.783 (0.754, 0.813)	–	–
Group 2	0.744 (0.715, 0.773)	− 0.039 (− 0.081, 0.002; 0.061)	–
Group 3	0.779 (0.751, 0.807)	− 0.005 (− 0.045, 0.036; 0.822)	0.035 (− 0.006, 0.075; 0.091)

Groups 1, 2, and 3 comprised the two questionnaire types having the EQ-5D-5L in the first, second, and last places, respectively

CI, confidence interval; US, the United States

The rank correlation only between the EQ-5D-5L index and the EORTC QLQ-C30’s role functioning subscale demonstrated significant differences between the EQF and QEF groups (difference 0.11; Additional file 1: Table S6). The rank correlations between the EQ-5D-5L’s five questions and the EORTC QLQ-C30 and FACT-G subscales have been presented in Additional file 1: Tables S7–S11.

The proportions of the patients with incomplete EQ-5D-5L in Groups 1, 2, and 3 were 0.11 (95% CI 0.08, 0.14), 0.11 (0.08, 0.14), and 0.05 (0.03, 0.07), respectively (Table 4). It was the lowest in Group 3. The difference in the proportions between Groups 3 and 1 was − 0.06 (95% CI − 0.10, − 0.02; p = 0.003), while that between Groups 3 and 2 was − 0.06 (− 0.10, − 0.02; 0.003). The analysis adjusted for covariates showed almost identical results (Additional file 1: Table S12). Both of the proportions of the patients with missing subscales of the FACT-G and those of the EORTC QLQ-C30 were lowest in Group 3 (Additional file 1: Tables S13 and S14).

**Table 4:** Proportions of the incomplete EQ-5D-5L in the three groups

	Proportion (95% CI)	Difference (95% CI; P-value)	
		vs Group 1	vs Group 2
Incomplete EQ-5D-5L for any reasons			
Group 1	0.11 (0.08, 0.14)	–	–
Group 2	0.11 (0.08, 0.14)	0.00 (− 0.05, 0.05; 0.978)	–
Group 3	0.05 (0.03, 0.07)	− 0.06 (− 0.10, − 0.02; 0.003)	− 0.06 (− 0.10, − 0.02; 0.003)
Did not return to the data center			
Group 1	0.05 (0.03, 0.08)	–	–
Group 2	0.06 (0.04, 0.09)	0.01 (− 0.03, 0.04; 0.668)	–
Group 3	0.02 (0.01, 0.04)	− 0.03 (− 0.06, 0.00; 0.038)	− 0.04 (− 0.07, − 0.01; 0.012)
Returned the questionnaire without any response			
Group 1	0.02 (0.00, 0.03)	–	–
Group 2	0.02 (0.01, 0.04)	0.01 (− 0.02, 0.03; 0.616)	–
Group 3	0.01 (0.00, 0.03)	0.00 (− 0.02, 0.02; 0.721)	− 0.01 (− 0.03, 0.01; 0.390)
Incomplete EQ-5D-5L with some responses to the returned questionnaire			
Group 1	0.04 (0.02, 0.06)	–	–
Group 2	0.03 (0.01, 0.04)	− 0.01 (− 0.04, 0.01; 0.360)	–
Group 3	0.01 (0.00, 0.02)	− 0.03 (− 0.05, 0.00; 0.023)	− 0.01 (− 0.03, 0.01; 0.156)

Groups 1, 2, and 3 contained the two questionnaire types that had the EQ-5D-5L in the first, second, and last places, respectively

CI, confidence interval

## Discussion

This study investigated the impact of the preceding cancer-specific HRQOL instruments on the subsequent EQ-5D-5L’s responses. Regarding the mobility question, the answers differed between the groups with the EQ-5D-5L placed first and second or third. The responses to the usual activities question varied between those with the EQ-5D-5L positioned first and second. As compared to the mean EQ-5D-5L indexes of the former, those of the latter were lower. Few correlation coefficients between the EQ-5D-5L index and the subscales of disease-specific HRQOL instruments differed between the groups with the EQ-5D-5L positioned first and second. The patients in the group with the EQ-5D-5L placed last tended to complete the EQ-5D-5L.

### Responses to EQ-5D-5L

The assimilation and contrast effects may explain the order effects on the EQ-5D-5L responses. If respondents answered a specific question before a general one, they may interpret the latter as having a similar meaning to the former, which is called the assimilation effect [25, 39]. However, if they are aware of the preceding specific question while answering the subsequent general one, they may infer the latter excluding the meaning of the former to avoid the redundancy of responses, which is known as the contrast effect [25, 39]. The main differences among the orders were observed in response levels of one (“no problems”) and two (“slight problems”). The patients in the group with the EQ-5D-5L placed second or last would have considered many situations before answering it to respond to the specific questions in the disease-specific HRQOL instruments; moreover, in this process, they may recall any problem relating to the EQ-5D-5L questions. Therefore, the assimilation effect of the specific questions on the EQ-5D-5L responses may have resulted in a small and a large proportion of “no problems” and “slight problems” reported, respectively. However, when two disease-specific HRQOL instruments preceded, the repetition of questions overlapping with each other may have rendered the patients more aware of the specific questions’ content; this may have caused a contrast effect due to the preceding question’s awareness.

For example, regarding usual activities, the specific questions’ contrast effect may help in explaining the difference in the responses between the groups with the EQ-5D-5L placed second and last; this is because most questions that would be related to usual activities overlap between the FACT-G and the EORTC QLQ-C30, such as GF1, GF2, and Q6; GF6 and Q7; and GP3 and Q26 (Table 5). Nevertheless, most specific questions related to mobility were only in the EORTC QLQ-C30, such as Q1, Q2, and Q3. Thus, the patients may have a likelihood of being unaware of these specific questions while answering the EQ-5D-5L’s mobility item; therefore, less contrast effect may occur. This may have resulted in a small proportion of “no problems” reported on the mobility questions in not only the group with the EQ-5D-5L placed second, but also the one where it was positioned last. Regarding anxiety/depression, similar to usual activities, majority of the specific questions would overlap between the FACT-G and the EORTC QLQ-C30, such as GE4, Q21, and Q23; GE5, GE6, and Q22; GE1, GE3, and Q24, although those of the former would be more detailed than those of the latter. Consequently, a small proportion of “no problems” may have been indicated regarding anxiety/depression only in the group with the EQ-5D-5L placed second, though the differences were not significant. Regarding self-care and pain/discomfort, limited specific questions were associated with the self-care or pain/discomfort items. This may explain the absence of discrepancies in these questions’ responses among the instrument orders.

**Table 5:**
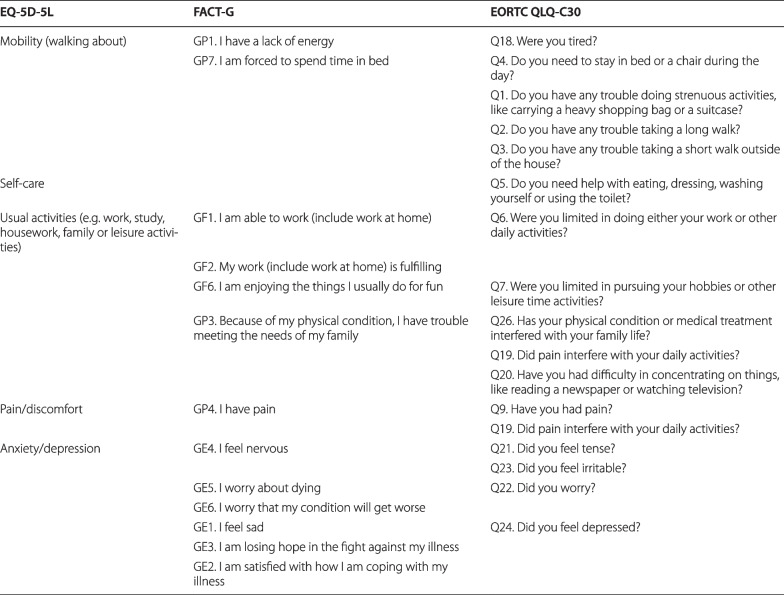
Questions in the FACT-G or the EORTC QLQ-C30 that may be related to the EQ-5D-5L

The question of the FACT-G or the EORTC QLQ-C30 should overlap with those of the other instrument in the same line

EORTC QLQ-C30, European Organization for Research and Treatment of Cancer Quality of Life Questionnaire Core 30; FACT-G, Functional Assessment of Cancer Therapy General

One of the previous studies has examined and found assimilation effect of specific health questions on self-rated health question [21]. This would be consistent with the above explanation.

### EQ-5D-5L index

We focused on the EQ-5D-5L index based on the Japanese value set, because the mean differences in the EQ-5D-5L’s index were similar among the three value sets. Thus far, no prior research has estimated the minimally important difference (MID) of the EQ-5D-5L index in cancer. Hence, we briefly estimated it for interpretation using the completed EQ-5D-5L population. In the distribution-based approach, 0.3 and 0.5 standard deviations are often used [40], and they were 0.055 (95% CI 0.052, 0.057) and 0.091 (0.087, 0.096), respectively. In the anchor-based approach, the performance status is employed frequently [40], and in cross-sectional studies, the differences between groups having varying anchors are utilized [41]. In our research, the difference between the ECOG performance status of 0 and 1 was 0.082 (95% CI 0.063, 0.102). Thus, the difference in the mean EQ-5D-5L indexes between the instrument orders was smaller than these values; furthermore, its 95% lower confidence limit was located among these values. These results suggest that the order effects on the EQ-5D-5L index would not be considerably larger than MID. However, considering the potential application of the EQ-5D-5L index to a long duration, the degree to which the instrument order affects the cost-effectiveness analysis is uncertain and should be evaluated.

### Correlation

Few correlation coefficients between the EQ-5D-5L index and the subscales of disease-specific HRQOL instruments differed among the instrument orders. This finding suggests that only a small assimilation or contrast effect existed; alternatively, both these effects existed in the population, however, mutually nullified the discrepancy in the correlation. In either case, the order effects on the coefficients of the subscales in regression models for mapping algorithms were considered to be limited.

### HRQOL instruments with missing values

The reasons why the EQ-5D-5L indexes, subscales of the FACT-G and those of the EORTC QLQ-C30 tended to be missing when the EQ-5D-5L was placed last are not clear. However, the first possible reason is that answering the EQ-5D-5L’s general questions may be easier after responding to the disease-specific HRQOL instruments’ specific ones than before answering them. The second possible reason is that patients may tend to decide to respond to the questionnaire after looking over the questionnaire with the EQ-5D-5L placed last. This is because the last instrument may be the instrument recalled mainly in the decision-making process, since in psychology it is known that, in free recall-task, the last item in a word list is easier to be recalled than other items (called recency effect) [42, 43]. Although the tendency of the first item in the list to be recalled (called primacy effect) is also known, a main cause of the primacy effect would be rehearsal of the item [42]. Contrary to the free-recall task, patients would not rehearse HRQOL instruments to memorize it; thus, primacy effect may not have occurred in the decision-making process. Therefore, the decision may be based largely on the burden of responding to the last HRQOL instrument. It might be recommended that the EQ-5D-5L should be placed at the last of questionnaires.

### Limitation

This study has certain limitations. First, the questionnaire booklets’ assignment to the patients was quasi-randomized and not strictly randomized with random numbers generated. Additionally, the medical staff could identify the questionnaire booklet types if they looked inside them. Thus, we cannot dismiss the possibility that they distributed the questionnaire booklets in an order different from instruction. However, they had no motivation to allocate these questionnaire booklets selectively to the patients with specific characteristics. Indeed, the patients’ observed characteristics were not unbalanced. Second, the patients might not have answered from the questionnaire booklet’s start, thus resulting in the order effects’ underestimation. Third, the order effects’ impact on the EQ-5D-5L may be different from this study when more severe patients or other disease-specific instruments are examined.

## Conclusions

In the patients undergoing drug therapy for advanced cancer, the preceding cancer-specific HRQOL instruments were found to impact the mobility and usual activities questions in the EQ-5D-5L. This resulted in a difference in the EQ-5D-5L index; however, our findings indicated that the difference size would not be considerably larger than the MID. Few correlation coefficients between the EQ-5D-5L index and the subscales of the disease-specific HRQOL instruments varied among the instrument orders. The patients tended to complete the EQ-5D-5L when it was placed at the end of the questionnaire.

## Supplementary Information

**Additional file 1.** Supplementary methods and tables.

## Data Availability

The data that support this study’s findings are available from the Center for Outcomes Research and Economic Evaluation for Health, National Institute of Public Health; however, restrictions apply to their availability, which were used under license for the current study. Hence, they are not publicly available.

## Abbreviations

ECOG: Eastern Cooperative Oncology Group
EORTC QLQ-C30: European Organization for Research and Treatment of Cancer Quality of Life Questionnaire Core 30
ePRO: Electric patient-reported outcome
FACT-G: Functional Assessment of Cancer Therapy General
HRQOL: Health-related quality of life
IPW: Inverse probability weighting
MID: Minimally important difference
QALY: Quality-adjusted life year
QOL-MAC: Quality of Life Mapping Algorithm for Cancer
