# Supplementary material for: Impacts of the preceding cancer-specific health-related quality of life instruments on the responses to the subsequent EQ-5D-5L
Source: Health Qual Life Outcomes. 2023 Jan 17;21:3. doi: 10.1186/s12955-022-02085-8 (PMC9843919; doi:10.1186/s12955-022-02085-8)
Supplement: Supplementary file 1 — Additional file 1. Supplementary methods and tables. [file 12955_2022_2085_MOESM1_ESM.pdf]

**Appendix for "Impacts of the Preceding Cancer-Specific Health-Related Quality of Life Measures on the Responses to the Subsequent EQ-5D-5L" in Health and Quality of Life Outcomes**

Shoki Izumi[1*], B.S., Yasuhiro Hagiwara[2*], Ph.D., M.P.H., Yutaka Matsuyama[2], Ph.D., Takeru Shiroiwa[3], Ph.D., M.P.H., Naruto Taira[4], M.D., Ph.D., Takuya Kawahara[5], Ph.D., M.P.H., Keiko Konomura[3], Ph.D., Shinichi Noto[6], Ph.D., Takashi Fukuda[3], Ph.D., Kojiro Shimozuma[7], M.D., Ph.D.

[1] Biostatistics and Bioinformatics Course, Graduate School of Interdisciplinary Information Studies, The University of Tokyo, Tokyo, Japan

[2] Department of Biostatistics, School of Public Health, The University of Tokyo, Tokyo, Japan

[3] Center for Outcomes Research and Economic Evaluation for Health, National Institute of Public Health, Wako, Japan

[4] Department of Breast and Endocrine Surgery, Okayama University Hospital, Okayama, Japan

[5] Clinical Research Promotion Center, The University of Tokyo Hospital, Tokyo, Japan

[6] Center for Health Economics & QOL Research, Niigata University of Health and Welfare, Niigata, Japan

[7] Department of Biomedical Sciences, College of Life Sciences, Ritsumeikan University, Kusatsu, Japan

**Correspondence to:**
Yasuhiro Hagiwara, Ph.D.
Department of Biostatistics, School of Public Health, The University of Tokyo
hagiwara@epistat.m.u-tokyo.ac.jp

**Supplementary Methods**

Analysis of EQ-5D-5L index

We assessed the mean EQ-5D-5L indexes of the subgroups using an analysis of variance with the order of the EQ-5D-5L, each covariate, and their interaction as explanatory variables. The p-values for the interaction terms were calculated using the type three sum of squares.

Using analysis of variance with the EQ-5D-5L's order, performance status, age, sex, tumor type, hospitalization, and adverse events at enrollment as the additional explanatory variable, the differences in the mean EQ-5D-5L indexes between the groups were estimated.

We conducted analysis of variance weighted by the inverse probability of observing the EQ-5D-5L index in order to adjust the impact of the missing one. The probability was estimated using linear logistic model with the order of the EQ-5D-5L, performance status, age, sex, tumor type, hospitalization, and adverse events at enrollment as explanatory variables. The 95% confidence intervals were constructed based on a conservative robust sandwich variance estimator.

The Spearman rank correlation coefficients between the EQ-5D-5L index and the subscales of EORTC QLQ-C30 and FACT-G were computed in the EQF and QEF and the EFQ and FEQ groups, respectively. The differences in the correlation coefficients among the groups were calculated; the p-values were determined using the Fisher z-transformation.

Analysis of proportion of HRQOL with missing subscales

Using linear binomial regression (linear probability model) with the EQ-5D-5L's order, performance status, age, sex, hospitalization, and adverse events at enrollment as the additional explanatory variable, the differences in the proportions of those patients whose EQ-5D-5L were incomplete between the groups were estimated, although compared to multivariable analysis of EQ-5D-5L indexes, tumor type was not added to the model because it did not converge.

The differences in the proportions of the patients with any of the FACT-G subscales or its total score not calculated between the groups were estimated using linear binomial regression with the EQ-5D-5L's order as the explanatory variable.

The differences in the proportions of the patients with any of the EORTC QLQ-C30 subscales not calculated between the groups were estimated using linear binomial regression with the EQ-5D-5L's order as the explanatory variable.

**Supplementary Results**

Table S1. Patient characteristics between the patients with complete and incomplete EQ-5D-5L.

| Characteristic | Completed EQ-5D-5LN = 937 | Incomplete EQ-5D-5LN = 92 |
|---|---|---|
| Age (in years) | 68 (58-74) | 65 (53-74) |
| Sex | | |
| Male | 509 (54%) | 44 (48%) |
| Female | 428 (46%) | 48 (52%) |
| Tumor type | | |
| Lung cancer | 329 (35%) | 15 (16%) |
| Stomach cancer | 66 (7%) | 12 (13%) |
| Colorectal cancer | 232 (25%) | 28 (30%) |
| Breast cancer | 116 (12%) | 19 (21%) |
| Other solid tumors | 194 (21%) | 18 (20%) |
| Stage at diagnosis | | |
| I | 49 (5%) | 5 (5%) |
| II | 74 (8%) | 4 (4%) |
| III | 192 (20%) | 20 (22%) |
| IV | 610 (65%) | 56 (61%) |
| Unknown | 12 (1%) | 7 (8%) |
| Site of metastasis or recurrence[a] | | |
| None | 83 (9%) | 12 (13%) |
| Liver | 211 (23%) | 25 (27%) |
| Lung | 296 (32%) | 29 (32%) |
| Bone | 169 (18%) | 24 (26%) |
| Brain | 100 (11%) | 2 (2%) |
| Lymph nodes | 373 (40%) | 31 (34%) |
| Others | 232 (25%) | 23 (25%) |
| History of surgery | | |
| Yes | 497 (53%) | 59 (64%) |
| No | 439 (47%) | 33 (36%) |
| Unknown | 1 (0%) | 0 (0%) |
| Hospitalization | | |
| Yes | 195 (21%) | 23 (25%) |

| | | |
|---|---|---|
| No | 742 (79%) | 69 (75%) |
| ECOG performance status | | |
| 0 | 464 (50%) | 47 (51%) |
| 1 | 385 (41%) | 34 (37%) |
| 2 | 67 (7%) | 7 (8%) |
| 3 | 20 (2%) | 3 (3%) |
| Unknown (0, 1, 2, or 3) | 1 (0%) | 1 (1%) |
| Type of treatment[a] | | |
| Chemotherapy | 606 (65%) | 62 (67%) |
| Endocrine therapy | 67 (7%) | 13 (14%) |
| Molecular targeted therapy | 163 (17%) | 4 (4%) |
| Immunotherapy | 112 (12%) | 9 (10%) |
| Palliative therapy | 37 (4%) | 5 (5%) |
| Others | 8 (1%) | 2 (2%) |
| Adverse event at enrollment | | |
| Yes | 606 (65%) | 59 (64%) |
| No | 329 (35%) | 32 (35%) |
| Unknown | 2 (0%) | 1 (1%) |

The median (IQR) and the number (%) were reported for age and other characteristics, respectively.

[a]Multiple choices were allowed.

ECOG, Eastern Cooperative Oncology Group; IQR, interquartile range.

Table S2. Responses to the five items in the EQ-5D-5L in Group 1, FEQ and QEF.

| | Response level | | | | | | |
|---|---|---|---|---|---|---|---|
| | 1 | 2 | 3 | 4 | 5 | vs Group 1 | vs FEQ |
| Mobility | | | | | | | |
| Group 1 | 194 (65%) | 51 (17%) | 26 (9%) | 23 (8%) | 6 (2%) | – | – |
| FEQ | 75 (50%) | 45 (30%) | 20 (13%) | 8 (5%) | 3 (2%) | 0.015 | – |
| QEF | 71 (46%) | 43 (28%) | 21 (14%) | 13 (8%) | 7 (5%) | <0.001 | 0.266 |
| Self-care | | | | | | | |
| Group 1 | 243 (81%) | 28 (9%) | 16 (5%) | 8 (3%) | 5 (2%) | – | – |
| FEQ | 124 (82%) | 17 (11%) | 5 (3%) | 4 (3%) | 1 (1%) | 0.686 | – |
| QEF | 118 (76%) | 23 (15%) | 7 (5%) | 1 (1%) | 6 (4%) | 0.274 | 0.192 |
| Usual activities | | | | | | | |
| Group 1 | 150 (50%) | 92 (31%) | 31 (10%) | 22 (7%) | 5 (2%) | – | – |
| FEQ | 66 (44%) | 55 (36%) | 16 (11%) | 10 (7%) | 4 (3%) | 0.308 | – |
| QEF | 56 (36%) | 68 (44%) | 16 (10%) | 6 (4%) | 9 (6%) | 0.024 | 0.292 |
| Pain/discomfort | | | | | | | |
| Group 1 | 122 (41%) | 126 (42%) | 39 (13%) | 10 (3%) | 3 (1%) | – | – |
| FEQ | 55 (36%) | 73 (48%) | 16 (11%) | 5 (3%) | 2 (1%) | 0.650 | – |
| QEF | 63 (41%) | 67 (43%) | 15 (10%) | 7 (5%) | 3 (2%) | 0.961 | 0.649 |
| Anxiety/depression | | | | | | | |
| Group 1 | 188 (63%) | 85 (28%) | 16 (5%) | 7 (2%) | 4 (1%) | – | – |
| FEQ | 81 (54%) | 51 (34%) | 15 (10%) | 3 (2%) | 1 (1%) | 0.064 | – |

| | | | | | | | |
|---|---|---|---|---|---|---|---|
| QEF | 88 (57%) | 49 (32%) | 9 (6%) | 7 (5%) | 2 (1%) | 0.196 | 0.639 |

Group 1 consisted of the two questionnaire types that had the EQ-5D-5L in the first position.

FEQ and QEF stand for the questionnaire types containing the HRQOL instruments in the order of FACT-G > EQ-5D-5L > EORTC QLQ-C30 and EORTC QLQ-C30 > EQ-5D-5L > FACT-G, respectively.

Table S3. Covariate-adjusted least square mean differences in the EQ-5D-5L indexes among the three groups.

| | Difference (95% CI; P-value) | |
|---|---|---|
| | vs Group 1 | vs Group 2 |
| Japanese value set | | |
| Group 1 | – | – |
| Group 2 | −0.028 (−0.052, −0.003; 0.026) | – |
| Group 3 | −0.007 (−0.031, 0.018; 0.59) | 0.021 (−0.003, 0.045; 0.083) |
| England value set | | |
| Group 1 | – | – |
| Group 2 | −0.021 (−0.048, 0.007; 0.135) | – |
| Group 3 | 0.001 (−0.026, 0.028; 0.929) | 0.022 (−0.005, 0.049; 0.106) |
| US value set | | |
| Group 1 | – | – |
| Group 2 | −0.028 (−0.062, 0.007; 0.112) | – |
| Group 3 | −0.004 (−0.038, 0.030; 0.812) | 0.024 (−0.010, 0.058; 0.166) |

Groups 1, 2, and 3 comprised the two questionnaire types having the EQ-5D-5L in the first, second, and last places, respectively.
CI, confidence interval; US, the United States.

Table S4. Mean EQ-5D-5L indexes adjusted for the missing EQ-5D-5L index through an inverse probability weighting.

| | | Difference (95% CI; P-value) | |
|---|---|---|---|
| | Mean (95% CI) | vs Group 1 | vs Group 2 |
| Japanese value set | | | |
| Group 1 | 0.796 (0.774, 0.818) | – | – |
| Group 2 | 0.760 (0.739, 0.781) | −0.036 (−0.067, −0.006; 0.021) | – |
| Group 3 | 0.788 (0.771, 0.806) | −0.007 (−0.036, 0.021; 0.606) | 0.029 (0.001, 0.056; 0.043) |
| England value set | | | |
| Group 1 | 0.820 (0.796, 0.844) | – | – |
| Group 2 | 0.790 (0.766, 0.815) | −0.029 (−0.064, 0.005; 0.096) | – |
| Group 3 | 0.821 (0.802, 0.841) | 0.001 (−0.030, 0.033; 0.926) | 0.031 (0.000, 0.062; 0.054) |
| US value set | | | |
| Group 1 | 0.783 (0.752, 0.813) | – | – |
| Group 2 | 0.744 (0.713, 0.775) | −0.039 (−0.082, 0.005; 0.082) | – |
| Group 3 | 0.778 (0.753, 0.803) | −0.004 (−0.044, 0.035; 0.829) | 0.034 (−0.005, 0.074; 0.089) |

Groups 1, 2, and 3 comprised the two questionnaire types that had the EQ-5D-5L in the first, second, and last places, respectively.

CI, confidence interval; US, the United States.

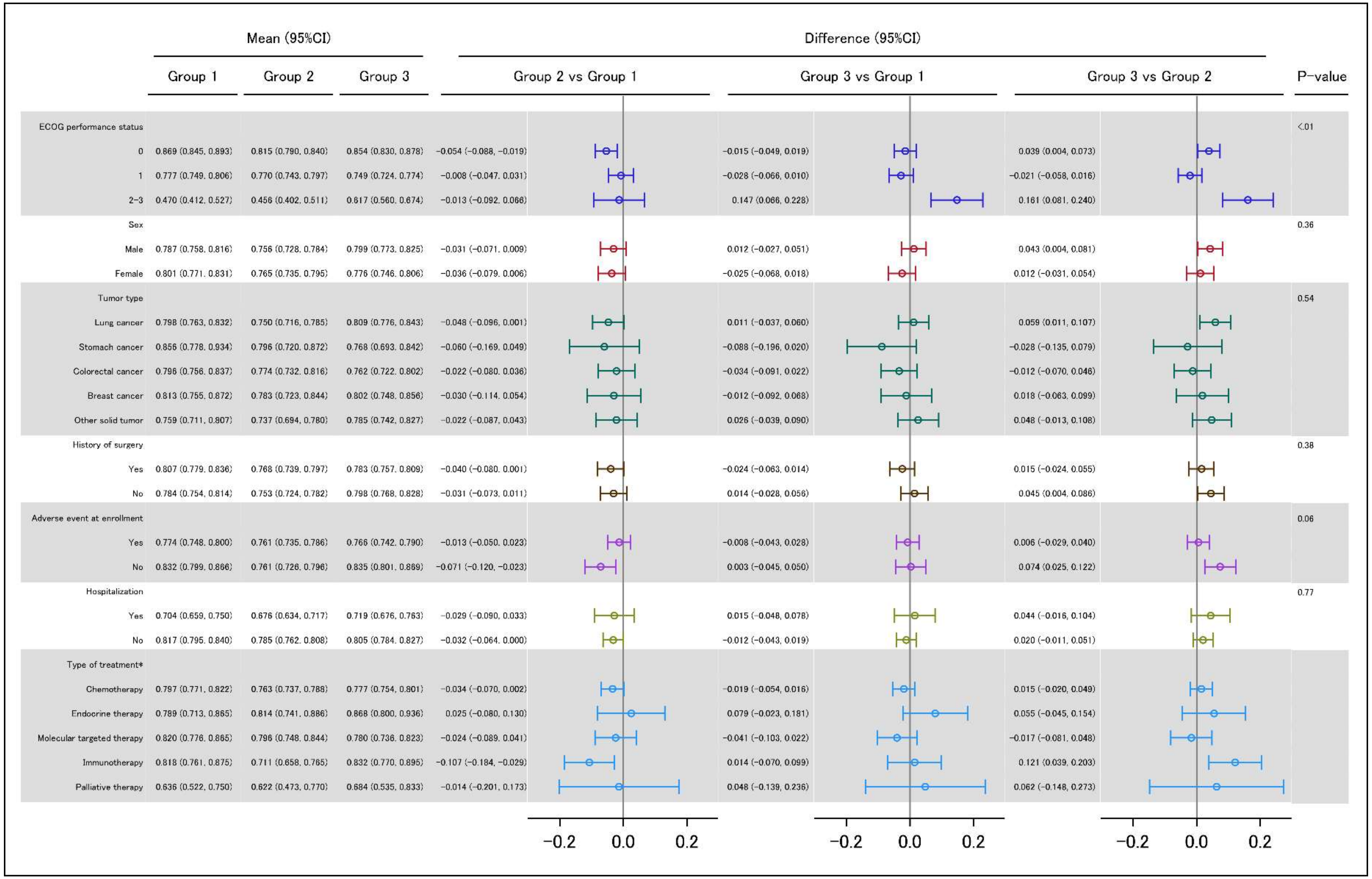


**Fig. S1** Forest plot for the subgroup analysis of the mean EQ-5D-5L index based on the value set for Japan

Groups 1, 2, and 3 consisted of the two questionnaire types that had the EQ-5D-5L in the first, second, and last places, respectively.

CI, confidence interval; ECOG, Eastern Cooperative Oncology Group.

*Multiple choices were allowed; thus, the p-value for the interaction term was not calculated.

Table S5. Mean EQ-5D-5L indexes in Group 1, FEQ and QEF.

| | Mean (95% CI) | Difference (95% CI; P-value) | |
|---|---|---|---|
| | | vs Group 1 | vs FEQ |
| Japanese value set | | | |
| Group 1 | 0.796 (0.776, 0.817) | – | – |
| FEQ | 0.770 (0.741, 0.799) | −0.026(−0.062, 0.009; 0.146) | – |
| QEF | 0.751 (0.722, 0.779) | −0.046(−0.081, −0.01; 0.011) | −0.019 (−0.060, 0.022; 0.352) |
| England value set | | | |
| Group 1 | 0.821 (0.798, 0.844) | – | – |
| FEQ | 0.805 (0.772, 0.837) | −0.016 (−0.056, 0.024; 0.427) | – |
| QEF | 0.777 (0.745, 0.809) | −0.044 (−0.084, −0.004; 0.031) | −0.028 (−0.074, 0.018; 0.238) |
| US value set | | | |
| Group 1 | 0.784 (0.754, 0.813) | – | – |
| FEQ | 0.761 (0.720, 0.802) | −0.022 (−0.073, 0.028; 0.385) | – |
| QEF | 0.727 (0.687, 0.768) | −0.056 (−0.106, −0.006; 0.028) | −0.034 (−0.092, 0.024; 0.254) |

Group 1 contained the two questionnaire types that had the EQ-5D-5L in the first position.

FEQ and QEF indicate the questionnaire types containing the HRQOL instruments in the order of FACT-G > EQ-5D-5L > EORTC QLQ-C30 and EORTC QLQ-C30 > EQ-5D-5L > FACT-G, respectively.

CI, confidence interval; US, the United States.

Table S6. Rank correlation between the EORTC QLQ-C30 and the FACT-G subscales and the EQ-5D-5L index based on the Japanese value set.

| | EORTC QLQ-C30 analysis | | | FACT-G analysis | | |
|---|---|---|---|---|---|---|
| | EQF | QEF | Difference (P-value) | EFQ | FEQ | Difference (P-value) |
| EORTC QLQ-C30 | | | | | | |
| Physical functioning | 0.75 | 0.71 | 0.04 (0.437) | – | – | – |
| Role functioning | 0.79 | 0.69 | 0.11 (0.042) | – | – | – |
| Emotional functioning | 0.41 | 0.39 | 0.02 (0.844) | – | – | – |
| Cognitive functioning | 0.41 | 0.48 | −0.07 (0.433) | – | – | – |
| Social functioning | 0.55 | 0.55 | −0.01 (0.927) | – | – | – |
| Global health status | 0.66 | 0.57 | 0.09 (0.215) | – | – | – |
| Fatigue | −0.71 | −0.66 | −0.06 (0.351) | – | – | – |
| Nausea and vomiting | −0.27 | −0.32 | 0.05 (0.638) | – | – | – |
| Pain | −0.63 | −0.57 | −0.07 (0.376) | – | – | – |
| Dyspnea | −0.44 | −0.46 | 0.02 (0.844) | – | – | – |
| Insomnia | −0.44 | −0.34 | −0.10 (0.299) | – | – | – |
| Appetite loss | −0.42 | −0.4 | −0.02 (0.853) | – | – | – |
| Constipation | −0.29 | −0.23 | −0.05 (0.617) | – | – | – |
| Diarrhea | −0.12 | −0.18 | 0.06 (0.599) | – | – | – |
| Financial difficulties | −0.38 | −0.33 | −0.05 (0.616) | – | – | – |
| FACT-G | | | | | | |
| Physical well-being | – | – | – | 0.73 | 0.69 | 0.04 (0.525) |
| Social/family well-being | – | – | – | 0.09 | 0.07 | 0.02 (0.861) |

| | | | | | | |
|---|---|---|---|---|---|---|
| Emotional well-being | – | – | – | 0.55 | 0.47 | 0.08 (0.364) |
| Functional well-being | – | – | – | 0.61 | 0.55 | 0.07 (0.397) |

EQF, QEF, EFQ, and FEQ stand for the questionnaire type containing the HRQOL instruments in the order of EQ-5D-5L > EORTC QLQ-C30 > FACT-G, EORTC QLQ-C30 > EQ-5D-5L > FACT-G, EQ-5D-5L > FACT-G > EORTC QLQ-C30, and FACT-G > EQ-5D-5L > EORTC QLQ-C30, respectively.

EORTC QLQ-C30, European Organization for Research and Treatment of Cancer Quality of Life Questionnaire Core 30; FACT-G, Functional Assessment of Cancer Therapy General.

Table S7. Rank correlation between the EORTC QLQ-C30 and the FACT-G subscales and the mobility question in the EQ-5D-5L.

| | EORTC QLQ-C30 analysis | | | FACT-G analysis | | |
|---|---|---|---|---|---|---|
| | EQF | QEF | Difference (P-value) | EFQ | FEQ | Difference (P-value) |
| EORTC QLQ-C30 | | | | | | |
| Physical functioning | −0.69 | −0.68 | 0.00 (0.938) | – | – | – |
| Role functioning | −0.67 | −0.56 | −0.11 (0.113) | – | – | – |
| Emotional functioning | −0.19 | −0.16 | −0.02 (0.835) | – | – | – |
| Cognitive functioning | −0.31 | −0.33 | 0.02 (0.828) | – | – | – |
| Social functioning | −0.43 | −0.41 | −0.02 (0.871) | – | – | – |
| Global health status | −0.53 | −0.45 | −0.08 (0.379) | – | – | – |
| Fatigue | 0.54 | 0.53 | 0.02 (0.838) | – | – | – |
| Nausea and vomiting | 0.23 | 0.31 | −0.08 (0.472) | – | – | – |
| Pain | 0.43 | 0.37 | 0.06 (0.528) | – | – | – |
| Dyspnea | 0.39 | 0.37 | 0.03 (0.776) | – | – | – |
| Insomnia | 0.32 | 0.26 | 0.06 (0.570) | – | – | – |
| Appetite loss | 0.31 | 0.34 | −0.03 (0.804) | – | – | – |
| Constipation | 0.26 | 0.12 | 0.14 (0.213) | – | – | – |
| Diarrhea | 0.02 | 0.22 | −0.20 (0.071) | – | – | – |
| Financial difficulties | 0.25 | 0.2 | 0.05 (0.629) | – | – | – |
| FACT-G | | | | | | |
| Physical well-being | – | – | – | −0.51 | −0.5 | −0.01 (0.890) |
| Social/family well-being | – | – | – | −0.08 | −0.17 | 0.09 (0.462) |
| Emotional well-being | – | – | – | −0.23 | −0.14 | −0.08 (0.467) |

| | | | | | | |
|---|---|---|---|---|---|---|
| Functional well-being | – | – | – | −0.4 | −0.43 | 0.03 (0.782) |

EQF, QEF, EFQ, and FEQ indicate the questionnaire types containing the HRQOL instruments in the order of EQ-5D-5L > EORTC QLQ-C30 > FACT-G, EORTC QLQ-C30 > EQ-5D-5L > FACT-G, EQ-5D-5L > FACT-G > EORTC QLQ-C30, and FACT-G > EQ-5D-5L > EORTC QLQ-C30, respectively.

EORTC QLQ-C30, European Organization for Research and Treatment of Cancer Quality of Life Questionnaire Core 30; FACT-G, Functional Assessment of Cancer Therapy General.

Table S8. Rank correlation between the EORTC QLQ-C30 and the FACT-G subscales and the self-care question in the EQ-5D-5L.

| | EORTC QLQ-C30 analysis | | | FACT-G analysis | | |
|---|---|---|---|---|---|---|
| | EQF | QEF | Difference (P-value) | EFQ | FEQ | Difference (P-value) |
| EORTC QLQ-C30 | | | | | | |
| Physical functioning | −0.59 | −0.37 | −0.22 (0.012) | – | – | – |
| Role functioning | −0.51 | −0.44 | −0.08 (0.397) | – | – | – |
| Emotional functioning | −0.19 | −0.17 | −0.02 (0.835) | – | – | – |
| Cognitive functioning | −0.26 | −0.34 | 0.08 (0.457) | – | – | – |
| Social functioning | −0.35 | −0.37 | 0.02 (0.842) | – | – | – |
| Global health status | −0.47 | −0.33 | −0.14 (0.143) | – | – | – |
| Fatigue | 0.43 | 0.31 | 0.13 (0.195) | – | – | – |
| Nausea and vomiting | 0.11 | 0.21 | −0.11 (0.341) | – | – | – |
| Pain | 0.36 | 0.35 | 0.01 (0.892) | – | – | – |
| Dyspnea | 0.27 | 0.19 | 0.08 (0.477) | – | – | – |
| Insomnia | 0.27 | 0.3 | −0.03 (0.811) | – | – | – |
| Appetite loss | 0.19 | 0.25 | −0.06 (0.590) | – | – | – |
| Constipation | 0.22 | 0.15 | 0.08 (0.487) | – | – | – |
| Diarrhea | −0.02 | 0.14 | −0.16 (0.153) | – | – | – |
| Financial difficulties | 0.27 | 0.16 | 0.12 (0.285) | – | – | – |
| FACT-G | | | | | | |
| Physical well-being | – | – | – | −0.38 | −0.38 | −0.01 (0.955) |
| Social/family well-being | – | – | – | −0.06 | −0.11 | 0.04 (0.711) |
| Emotional well-being | – | – | – | −0.25 | −0.18 | −0.08 (0.505) |

| | | | | | | | |
|---|---|---|---|---|---|---|---|
| Functional well-being | – | – | – | | −0.35 | −0.37 | 0.02 (0.843) |

EQF, QEF, EFQ, and FEQ indicate the questionnaire types containing the HRQOL instruments in the order of EQ-5D-5L > EORTC QLQ-C30 > FACT-G, EORTC QLQ-C30 > EQ-5D-5L > FACT-G, EQ-5D-5L > FACT-G > EORTC QLQ-C30, and FACT-G > EQ-5D-5L > EORTC QLQ-C30, respectively.

EORTC QLQ-C30, European Organization for Research and Treatment of Cancer Quality of Life Questionnaire Core 30; FACT-G, Functional Assessment of Cancer Therapy General.

Table S9. Rank correlation between the EORTC QLQ-C30 and the FACT-G subscales and the usual activities question in the EQ-5D-5L.

| | EORTC QLQ-C30 analysis | | | FACT-G analysis | | |
|---|---|---|---|---|---|---|
| | EQF | QEF | Difference (P-value) | EFQ | FEQ | Difference (P-value) |
| EORTC QLQ-C30 | | | | | | |
| Physical functioning | −0.75 | −0.66 | −0.09 (0.110) | – | – | – |
| Role functioning | −0.84 | −0.74 | −0.10 (0.015) | – | – | – |
| Emotional functioning | −0.29 | −0.33 | 0.04 (0.681) | – | – | – |
| Cognitive functioning | −0.41 | −0.41 | 0.00 (1.000) | – | – | – |
| Social functioning | −0.53 | −0.52 | −0.01 (0.868) | – | – | – |
| Global health status | −0.65 | −0.51 | −0.14 (0.058) | – | – | – |
| Fatigue | 0.68 | 0.64 | 0.04 (0.529) | – | – | – |
| Nausea and vomiting | 0.28 | 0.3 | −0.02 (0.859) | – | – | – |
| Pain | 0.55 | 0.44 | 0.11 (0.189) | – | – | – |
| Dyspnea | 0.4 | 0.48 | −0.07 (0.441) | – | – | – |
| Insomnia | 0.38 | 0.23 | 0.15 (0.162) | – | – | – |
| Appetite loss | 0.38 | 0.36 | 0.02 (0.821) | – | – | – |
| Constipation | 0.25 | 0.19 | 0.06 (0.594) | – | – | – |
| Diarrhea | 0.12 | 0.12 | 0.00 (1.000) | – | – | – |
| Financial difficulties | 0.43 | 0.33 | 0.09 (0.353) | – | – | – |
| FACT-G | | | | | | |
| Physical well-being | – | – | – | −0.68 | −0.6 | −0.08 (0.280) |
| Social/family well-being | – | – | – | 0.04 | −0.02 | 0.05 (0.667) |

| | | | | | | |
|---|---|---|---|---|---|---|
| Emotional well-being | – | – | – | −0.4 | −0.32 | −0.08 (0.446) |
| Functional well-being | – | – | – | −0.51 | −0.55 | 0.04 (0.665) |

EQF, QEF, EFQ, and FEQ denote the questionnaire types containing the HRQOL instruments in the order of EQ-5D-5L > EORTC QLQ-C30 > FACT-G, EORTC QLQ-C30 > EQ-5D-5L > FACT-G, EQ-5D-5L > FACT-G > EORTC QLQ-C30, and FACT-G > EQ-5D-5L > EORTC QLQ-C30, respectively.

EORTC QLQ-C30, European Organization for Research and Treatment of Cancer Quality of Life Questionnaire Core 30; FACT-G, Functional Assessment of Cancer Therapy General.

Table S10. Rank correlation between the EORTC QLQ-C30 and the FACT-G subscales and the pain/discomfort question in the EQ-5D-5L.

| | EORTC QLQ-C30 analysis | | | FACT-G analysis | | |
|---|---|---|---|---|---|---|
| | EQF | QEF | Difference (P-value) | EFQ | FEQ | Difference (P-value) |
| EORTC QLQ-C30 | | | | | | |
| Physical functioning | −0.49 | −0.37 | −0.11 (0.228) | – | – | – |
| Role functioning | −0.51 | −0.41 | −0.10 (0.272) | – | – | – |
| Emotional functioning | −0.37 | −0.29 | −0.08 (0.429) | – | – | – |
| Cognitive functioning | −0.36 | −0.37 | 0.02 (0.864) | – | – | – |
| Social functioning | −0.4 | −0.29 | −0.11 (0.297) | – | – | – |
| Global health status | −0.52 | −0.46 | −0.06 (0.493) | – | – | – |
| Fatigue | 0.61 | 0.43 | 0.19 (0.027) | – | – | – |
| Nausea and vomiting | 0.22 | 0.19 | 0.03 (0.766) | – | – | – |
| Pain | 0.74 | 0.71 | 0.04 (0.487) | – | – | – |
| Dyspnea | 0.39 | 0.36 | 0.03 (0.800) | – | – | – |
| Insomnia | 0.5 | 0.29 | 0.21 (0.031) | – | – | – |
| Appetite loss | 0.47 | 0.37 | 0.11 (0.269) | – | – | – |
| Constipation | 0.19 | 0.25 | −0.05 (0.627) | – | – | – |
| Diarrhea | 0.1 | 0.11 | −0.01 (0.925) | – | – | – |
| Financial difficulties | 0.28 | 0.26 | 0.02 (0.825) | – | – | – |
| FACT-G | | | | | | |
| Physical well-being | – | – | – | −0.67 | −0.58 | −0.09 (0.196) |
| Social/family well-being | – | – | – | 0 | −0.12 | 0.12 (0.302) |

| | | | | | | |
|---|---|---|---|---|---|---|
| Emotional well-being | – | – | – | −0.45 | −0.37 | −0.08 (0.427) |
| Functional well-being | – | – | – | −0.46 | −0.37 | −0.09 (0.350) |

EQF, QEF, EFQ, and FEQ indicate the questionnaire types containing the HRQOL instruments in the order of EQ-5D-5L > EORTC QLQ-C30 > FACT-G, EORTC QLQ-C30 > EQ-5D-5L > FACT-G, EQ-5D-5L > FACT-G > EORTC QLQ-C30, and FACT-G > EQ-5D-5L > EORTC QLQ-C30.

EORTC QLQ-C30, European Organization for Research and Treatment of Cancer Quality of Life Questionnaire Core 30; FACT-G, Functional Assessment of Cancer Therapy General.

Table S11. Rank correlation between the EORTC QLQ-C30 and the FACT-G subscales and the anxiety/depression question in the EQ-5D-5L.

| | EORTC QLQ-C30 analysis | | | FACT-G analysis | | |
|---|---|---|---|---|---|---|
| | EQF | QEF | Difference (P-value) | EFQ | FEQ | Difference (P-value) |
| EORTC QLQ-C30 | | | | | | |
| Physical functioning | −0.26 | −0.26 | 0.00 (0.994) | – | – | – |
| Role functioning | −0.28 | −0.22 | −0.07 (0.544) | – | – | – |
| Emotional functioning | −0.52 | −0.45 | −0.07 (0.419) | – | – | – |
| Cognitive functioning | −0.23 | −0.32 | 0.09 (0.397) | – | – | – |
| Social functioning | −0.33 | −0.33 | 0.00 (0.982) | – | – | – |
| Global health status | −0.34 | −0.28 | −0.07 (0.530) | – | – | – |
| Fatigue | 0.3 | 0.34 | −0.04 (0.664) | – | – | – |
| Nausea and vomiting | 0.14 | 0.23 | −0.09 (0.423) | – | – | – |
| Pain | 0.29 | 0.26 | 0.02 (0.831) | – | – | – |
| Dyspnea | 0.2 | 0.23 | −0.03 (0.782) | – | – | – |
| Insomnia | 0.27 | 0.19 | 0.08 (0.476) | – | – | – |
| Appetite loss | 0.2 | 0.22 | −0.02 (0.892) | – | – | – |
| Constipation | 0.26 | 0.23 | 0.03 (0.778) | – | – | – |
| Diarrhea | 0.08 | 0.05 | 0.03 (0.796) | – | – | – |
| Financial difficulties | 0.21 | 0.28 | −0.08 (0.487) | – | – | – |
| FACT-G | | | | | | |
| Physical well-being | – | – | – | −0.52 | −0.48 | −0.04 (0.643) |
| Social/family well-being | – | – | – | −0.15 | 0.01 | −0.16 (0.175) |

| | | | | | | |
|---|---|---|---|---|---|---|
| Emotional well-being | – | – | – | −0.68 | −0.63 | −0.05 (0.499) |
| Functional well-being | – | – | – | −0.51 | −0.31 | −0.20 (0.044) |

EQF, QEF, EFQ, and FEQ denote the questionnaire types containing the HRQOL instruments in the order of EQ-5D-5L > EORTC QLQ-C30 > FACT-G, EORTC QLQ-C30 > EQ-5D-5L > FACT-G, EQ-5D-5L > FACT-G > EORTC QLQ-C30, and FACT-G > EQ-5D-5L > EORTC QLQ-C30, respectively.

EORTC QLQ-C30, European Organization for Research and Treatment of Cancer Quality of Life Questionnaire Core 30; FACT-G, Functional Assessment of Cancer Therapy General.

Table S12. Covariate-adjusted least square mean differences in proportions of the incomplete EQ-5D-5L among the three groups.

| | Difference (95% CI; P-value) | |
|---|---|---|
| | vs Group 1 | vs Group 2 |
| Incomplete EQ-5D-5L for any reasons | | |
| Group 1 | – | – |
| Group 2 | 0.00 (−0.04, 0.05; 0.922) | – |
| Group 3 | −0.05 (−0.09, −0.02; 0.007) | −0.06 (−0.10, −0.02; 0.006) |

Groups 1, 2, and 3 contained the two questionnaire types that had the EQ-5D-5L in the first, second, and last places, respectively.

CI, confidence interval.

Table S13. Proportions of the FACT-G with missing subscales in the three groups.

| | Proportion (95% CI) | Difference (95% CI; P-value) vs Group 1 | Difference (95% CI; P-value) vs Group 2 |
|---|---|---|---|
| FACT-G with any of the four subscales or its total score missing for any reasons | | | |
| Group 1 | 0.12 (0.09, 0.16) | – | – |
| Group 2 | 0.11 (0.07, 0.14) | −0.02 (−0.07, 0.03; 0.487) | – |
| Group 3 | 0.06 (0.03, 0.08) | −0.07 (−0.11, −0.02; 0.002) | −0.05 (−0.09, −0.01; 0.016) |
| Did not return to the data center | | | |
| Group 1 | 0.05 (0.03, 0.08) | – | – |
| Group 2 | 0.06 (0.04, 0.09) | 0.01 (−0.03, 0.04; 0.668) | – |
| Group 3 | 0.02 (0.01, 0.04) | −0.03 (−0.06, 0.00; 0.038) | −0.04 (−0.07, −0.01; 0.012) |
| Returned the questionnaire without any response | | | |
| Group 1 | 0.02 (0.00, 0.03) | – | – |
| Group 2 | 0.02 (0.01, 0.04) | 0.01 (−0.02, 0.03; 0.616) | – |
| Group 3 | 0.01 (0.00, 0.03) | 0.00 (−0.02, 0.02; 0.721) | −0.01 (−0.03, 0.01; 0.390) |
| FACT-G with any of the four subscales or its total score missing with some responses to the returned questionnaire | | | |
| Group 1 | 0.05 (0.03, 0.08) | – | – |
| Group 2 | 0.02 (0.01, 0.04) | −0.03 (−0.06, 0.00; 0.040) | – |
| Group 3 | 0.02 (0.01, 0.03) | −0.03 (−0.06, −0.01; 0.021) | 0.00 (−0.02, 0.02; 0.777) |

Groups 1, 2, and 3 contained the two questionnaire types that had the EQ-5D-5L in the first, second, and last places, respectively.
CI, confidence interval; FACT-G, Functional Assessment of Cancer Therapy General.

Table S14. Proportions of the EORTC QLQ-C30 with missing subscale in the three groups.

| | Proportion (95% CI) | Difference (95% CI; P-value) vs Group 1 | Difference (95% CI; P-value) vs Group 2 |
|---|---|---|---|
| EORTC QLQ-C30 with any of the fifteen subscales missing for any reasons | | | |
| Group 1 | 0.13 (0.09, 0.16) | – | – |
| Group 2 | 0.13 (0.09, 0.16) | 0.00 (−0.05, 0.05; 0.919) | – |
| Group 3 | 0.06 (0.03, 0.08) | −0.07 (−0.11, −0.03; 0.001) | −0.07 (−0.11, −0.02; 0.002) |
| Did not return to the data center | | | |
| Group 1 | 0.05 (0.03, 0.08) | – | – |
| Group 2 | 0.06 (0.04, 0.09) | 0.01 (−0.03, 0.04; 0.668) | – |
| Group 3 | 0.02 (0.01, 0.04) | −0.03 (−0.06, 0.00; 0.038) | −0.04 (−0.07, −0.01; 0.012) |
| Returned the questionnaire without any response | | | |
| Group 1 | 0.02 (0.00, 0.03) | – | – |
| Group 2 | 0.02 (0.01, 0.04) | 0.01 (−0.02, 0.03; 0.616) | – |
| Group 3 | 0.01 (0.00, 0.03) | 0.00 (−0.02, 0.02; 0.721) | −0.01 (−0.03, 0.01; 0.390) |
| EORTC QLQ-C30 with any of the fifteen subscales missing with some responses to the returned questionnaire | | | |
| Group 1 | 0.06 (0.03, 0.08) | – | – |
| Group 2 | 0.04 (0.02, 0.06) | −0.02 (−0.05, 0.02; 0.341) | – |
| Group 3 | 0.02 (0.01, 0.03) | −0.04 (−0.06, −0.01; 0.013) | −0.02 (−0.05, 0.00; 0.115) |

Groups 1, 2, and 3 contained the two questionnaire types that had the EQ-5D-5L in the first, second, and last places, respectively.
CI, confidence interval.
